# DNA Methylation Status of Regulatory Regions of Apoptosis-Associated Genes in Dystropy «Huntington’s Disease—Non-Small Cell Lung Cancer»

**DOI:** 10.3390/epigenomes9030028

**Published:** 2025-08-07

**Authors:** Nadezhda P. Babushkina, Elena Yu. Bragina, Densema E. Gomboeva, Iuliia A. Koroleva, Sergey N. Illarioshkin, Sergey A. Klyushnikov, Nataliya Yu. Abramycheva, Maria A. Nikitina, Valentina M. Alifirova, Nikolai V. Litviakov, Marina K. Ibragimova, Matvey M. Tsyganov, Irina A. Tsydenova, Aleksei A. Zarubin, Irina A. Goncharova, Maria V. Golubenko, Ramil R. Salakhov, Aleksei A. Sleptcov, Aksana N. Kucher, Maria S. Nazarenko, Valery P. Puzyrev

**Affiliations:** 1 Research Institute of Medical Genetics, Tomsk National Research Medical Center, Russian Academy of Sciences, 10 Ushayka Embankment, Tomsk 634050, Russia; elena.bragina@medgenetics.ru (E.Y.B.); maria.nazarenko@medgenetics.ru (M.S.N.);; 2 Research Center of Neurology, 80 Volokolamskoye Shosse, Moscow 125367, Russiasergeklyush@gmail.com (S.A.K.);; 3 Department of Neurology, Siberian State Medical University, 2 Moscow Tract, Tomsk 634012, Russia; nikitina.ma1@ssmu.ru (M.A.N.);; 4 Tomsk Cancer Research Institute, Tomsk National Research Medical Center, Russian Academy of Sciences, 5 Kooperativny Street, Tomsk 634009, Russia

**Keywords:** apoptosis-associated genes, CpG site, DNA methylation, dystropy, Huntington’s disease, lung cancer

## Abstract

**Background.** Studies of comorbid (syntropic) and inversely comorbid (rarely occurring together, i.e., dystropic) diseases have focused on the search for molecular causes of this phenomenon. **Materials.** We investigated DNA methylation levels in regulatory regions of 23 apoptosis-associated genes as candidate loci associated with the “cancer–neurodegeneration” dystropy in patients with Huntington’s disease (HD) and patients with non–small cell lung cancer (LC). **Results.** Statistically significant differences in methylation levels between the HD and LC groups were found for 41 CpG sites in 16 genes. The results show that five genes (*SETDB1*, *TWIST1*, *HDAC1*, *SP1*, and *GRIA2*) are probably involved in the phenomenon of inverse comorbidity of these diseases. For these genes, the methylation levels of the studied CpG sites were altered in opposite directions in the two groups of patients, compared to the control group. **Conclusions.** For the *SP1* gene, the above hypothesis is supported by our analysis of open-access data on gene expression in patients with the aforementioned diagnoses and fits a probable mechanism of the “HD–LC” dystropy.

## 1. Introduction

Research on comorbid (syntropic) and inversely comorbid (rarely occurring together, dystropic) diseases is widespread, aiming to identify the basic molecular causes of this phenomenon [1,2]. In this study, we use the term ‘dystropy’ to refer to the inverse comorbidity between two diseases.

Numerous epidemiological studies have shown that patients with neurodegenerative diseases, either multifactorial (e.g., Alzheimer’s disease or Parkinson’s disease) or monogenic (Huntington’s disease; HD), rarely develop cancer [3]. Complex interactions involving a wide range of biological processes contribute to the phenotypic “conflict” between cancers and neurodegenerative diseases. The specific processes involved vary depending on the disease pairs [4,5].

In this paper, we focused on HD and non–small cell lung cancer (LC), which rarely occur together [6]. HD is caused by the expansion of trinucleotide (CAG) repeats (36 or more) in exon 1 of the huntingtin (*HTT*) gene [7], which encodes a ubiquitously expressed regulatory protein.

Interestingly, normal huntingtin suppresses apoptosis, whereas mutant huntingtin promotes it [8]. Conversely, LC is characterized by the suppression of apoptosis [9]. We hypothesized that the regulation of the expression of genes involved in apoptotic processes may be one of the molecular mechanisms underlying the dystropy between HD and LC. Using bioinformatic approaches, our previous study identified apoptosis-related candidate genes associated with dystropy between HD and various cancer types [6,10].

DNA methylation levels in the regulatory regions of genes in peripheral blood leukocytes are useful markers for evaluating gene function at the organism level. The prognostic significance of these markers in various pathologies is being actively studied [11,12,13,14,15].

In this study, we examined DNA methylation levels in the regulatory regions of apoptosis-associated genes as candidate loci related to the dystropy of HD and LC (a dystropic or inversely comorbid relationship) [10]. We performed this analysis in the peripheral blood leukocytes of patients with HD or LC (Table S1).

## 2. Results

### 2.1. Characterization of Methylation Levels and Patterns at the Examined CpG Sites in Apoptosis-Associated Genes

**Figure 1:**
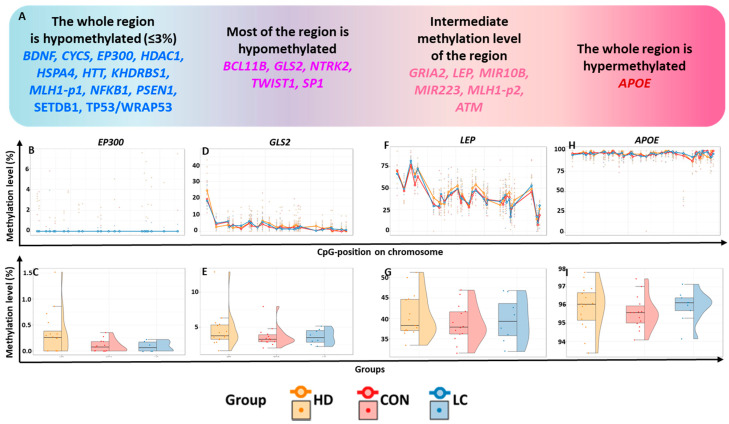
General characterization of the studied regions. (**A**) Classification of genes by methylation level of the studied regions; methylation levels (in %) in the regions of genes (**B**,**C**) *EP300*, (**D**,**E**) *GLS2*, (**F**,**G**) *LEP*, and (**H**,**I**) *APOE*. (**B**,**D**,**F**,**H**): at individual CpG sites; (**C**,**E**,**G**,**I**): in the whole region in peripheral blood leukocytes from nominally healthy individuals (CON) or patients with HD or LC.

We quantified the methylation of 596 CpG sites within 24 regulatory regions of 23 genes (Table S4). The general characteristics of these genomic regions were determined by averaging the methylation levels across all three study groups without considering the pathological status. DNA methylation levels varied significantly among these regions, ranging from 0 to 100% (Figure 1). The expression levels in whole blood differed among all the studied genes (Table S1).

Based on methylation status, the analyzed genes were tentatively subdivided into four groups (Figure 1A). Hypomethylated genes were defined as those with all studied CpG sites having a methylation level ≤3% ( 12 such genes), whereas hypermethylated genes had nearly complete methylation (~100%; 1 gene identified).

#### 2.1.1. Hypomethylated Genes

Most of the CpG sites with very low methylation levels (356 CpG sites) were located within 12 genes, which we will refer to as “hypomethylated genes” (Figure 1A–C, Table S4). For the genes *HTT*, *PSEN1*, *SETDB1*, *WRAP53*, and *EP300*, the studied regions encompassed most of the relevant CpG islands (CGIs) and contained at least one transcription start site (TSS) (the localization and structure of each TSS were determined using the Eukaryotic Promoter Database [16]).

The analyzed regions of genes *BDNF*, *HDAC1*, *CYCS*, and *NFKB1,* and one of the regions in the *MLH1* gene regulatory sequence (*MLH1*_p1) were found to be either fully or partially located within CGIs in the 5′-untranslated regions (5′-UTR) of these genes. These regions harbor regulatory sites but do not directly include the TSS. The CpGs studied for *HSPA4* and *KHDRBS1* are localized within CGIs downstream of the TSS in the region corresponding to the noncoding 5′ end of the transcript. These regions also contain binding sites for many transcription factors (TFs). In the majority of the “hypomethylated genes,” the analyzed regions are located in the CGIs, except for *CYCS*, *HDAC1*, *NFKB1*, *SETDB*, and *WRAP53*. In these latter genes, some of the hypomethylated sites (21 CpG sites in total) are situated within 200 bp of a CGI (in the near “shore” region).

#### 2.1.2. Genes Exhibiting Low Methylation Levels Across Most of the Analyzed Region

For the 26 CpG sites of the *GLS2* gene, the average methylation levels varied from 1.32%to 6.68%, whereas for one site (CpG_1), these levels reached 23.94% (Figure 1D,E, Table S4). All hypomethylated CpG sites were located within a CGI. The investigated region corresponded to the beginning of the *GLS2* transcript. The studied region of the *NTRK2* gene is located entirely within a CGI and overlaps with the regulatory regions of TSSs. This region is also predominantly hypomethylated (0.33–6.9% for 15 CpG sites, <3% for seven more CpG sites), but the methylation level of one site (CpG_11) was 10.22%. In the studied region of the *TWIST1* gene, the methylation level of 20 CpG sites was extremely low (0.39–4.59%); for eight CpG sites, it was in the range of 5.04–9.68%, and for one CpG site (CpG_4), the level reached 20.59%. The tested region covered the CGI shore and may harbor regulatory elements for the TSS of *TWIST1*.

The genes *BCL11B* and *SP1* also showed uneven methylation levels at the investigated sites. For instance, a very low methylation level (0.71–4.98%) was registered for 10 CpG sites in the examined region of the *BCL11B*; for 10 other CpG sites, the average methylation level was 5.25–14.00%; and for one site (CpG_1), it reached 20.67%. The studied sequence of this gene does not overlap with the TSS region (corresponding to the beginning of the transcript) but is predominantly located in a CGI, including all six hypomethylated sites.

For the *SP1* gene, we examined the CGI shores of its promoter. Seven sites had extremely low methylation levels (0.71–2.7%), while eight sites had slightly higher levels (6.27–25.60%) (Table S4).

#### 2.1.3. Genes Exhibiting Intermediate Methylation Levels in the Tested Region

Intermediate methylation levels (6.58–67.50%) were documented for CGIs in six genes: *GRIA2*, *LEP*, *MIR10B*, *MIR223*, *MLH1* ( *MLH1*-p2 fragment), and *ATM* (Table S4). Two microRNA genes were located within the studied sequences. *MIR10B* lies inside the CGI in the *HOXD* gene cluster, and *MIR223* is situated inside the GeneHancer promoter GH0XJ066006 near the *VSIG4* gene within exon 2 of its *MIR223GH*. The methylation levels of the 22 CpG sites in *MIR10B* ranged from 11.77% to 50.03%. Since this gene is located on chromosome X, it was examined separately in males and females. The methylation level range of the analyzed *MIR223* gene region was 11.51–43.72% for males and 13.71–67.50% for females (Table S4).

Among the 17 CpG sites in *GRIA2*, only one (CpG_1) showed a low methylation level (2.11%); at seven other sites, the methylation level ranged from 6.58% to 14.98%, and in the remaining nine sites, it ranged from 17.27% to 38.87% (Table S4). The region in question, located on the shore of two CGIs, is located at a locus corresponding to the beginning of the transcript and includes the entire first intron, partially encompassing exons 1 and 2.

The studied region of the *LEP* gene is located within a CGI in the regulatory region, near the TSS. Three subregions were identified in this region: one with a higher methylation level (CpG_1 to CpG_5, 45.89–77.72%), another with an intermediate methylation level (CpG_6 to CpG_25, 32.11–50.89%), and a third with a low methylation level (Figure 1F,G, Table S4).

The *MLH1*-p2 region is located on a CpG shore in a probable promoter regulatory element within the GeneHancer GH03J036988 promoter. The methylation levels of CpG sites in this region varied from 31.75% to 69.99% (Table S4).

The studied region for the *ATM* gene is rather distant from a CGI, located on a CpG shelf (a region within ±4000 bp of the CGI). The GeneHancer GH11J108220 enhancer was found within this region. It should be noted that all CpG sites in question are situated within this regulatory element and could be important for its functions. A high methylation level was registered for CpG_1 (94.96%), while for the sites CpG_2-CpG_6, the methylation level varied from 12.76% to 58.35% (Table S4).

#### 2.1.4. A Hypermethylated Region

The CGI in exon 4 of the *APOE* gene is located within the coding region. Only two sites in this gene had methylation levels less than 90%: CpG_29 (88.71%) and CpG_30 (88.68%). For the other sites, we documented levels between 90.21% and 98.48% (Figure 1H,I, Table S4).

### 2.2. Comparison of Methylation Levels for CpG Sites in Apoptosis-Associated Genes in Blood Leukocytes Between the Groups

We searched for differentially methylated DNA regions in HD patients compared to controls and LC patients compared to controls. CGIs and promoters typically exhibit very low DNA methylation levels with a narrow range of fluctuations [17], which is consistent with our results (Tables S4 and S7). Furthermore, evidence suggests that even a 1.5% difference in methylation levels in such regions can be substantial and may lead to alterations in gene expression [17,18,19,20,21]. Therefore, we established thresholds of a ≥ re, change in the methylation level, and a significance level *p* < 0.05 after correction for multiple comparisons (using the Benjamini–Hochberg method) as criteria for recognizing differential methylation.

**Figure 2:**
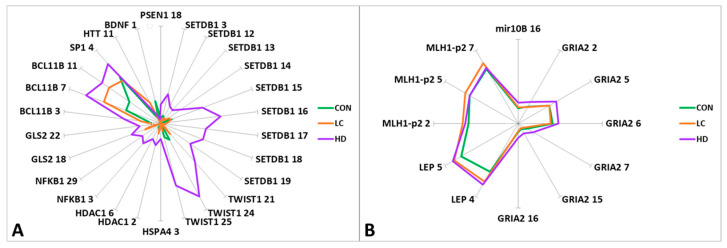
Graphical representation of the differences in methylation levels between HD patients, LC patients, and CON subjects. (**A**) CpG sites with methylation levels up to 10%; (**B**) CpG sites with methylation levels of 10% to 70%. The genes and ID of the CpG sites in the gene are indicated.

Statistically significant differences in methylation levels between the groups were found for 41 CpG sites in 16 genes (Figure 2, Table S7). We observed a general trend of higher methylation levels in blood leukocytes of the HD group than in controls and patients with LC for 33 CpG sites in 12 genes (*PSEN1*, *SETDB1*, *TWIST1*, *HSPA4*, *HDAC1*, *NFKB1*, *GLS2*, *MIR10B*, *SP1*, *GRIA2*, *BCL11B*, and *LEP*). Significant differences were detected in 26 CpG sites in these genes. Conversely, patients with LC exhibited methylation levels similar to those of the controls (CON), with statistically significant differences between the CON and LC groups for seven CpG sites (Table S7).

Statistically significant differences between the CON and HD groups were detected in the *APOE* region (CpG_11 and CpG_21), similar to the differences between the control and LC groups (CpG_11). However, we observed changes in the opposite direction for some CpG sites: for CpG_11 and CpG_36, the level followed the order CON > LC > HD, while for CpG_21, the direction of methylation changes was CON < LC < HD. In this region, 40 CpG sites were tested; however, it was not possible to identify blocks with unidirectional changes in the methylation levels of CpG sites. Thus, each group had its own “pattern” of methylation (Supplementary Table S3).

For two genes (*BCL11B* and *LEP*), we noted a marked increase in methylation in the order CON < LC < HD (Table S7).

For 20 CpG sites in eight genes, there were significant differences between the HD and LC groups (Table S7). When the two diseases were compared, the methylation levels of 15 CpG sites changed in the opposite direction compared to the control group. The most interesting genes were those that manifested statistically significant bidirectional changes in methylation level: *SETDB1* (CpG_3 and CpG_15), *TWIST1* (CpG_24 and CpG_25), *HDAC1* (CpG_6), *SP1* (CpG_4), and *GRIA2* (CpG_2, CpG_5, CpG_6, CpG_7, CpG_15, and CpG_16) (Table S7).

### 2.3. Functional Annotation of Regulatory Elements in the Studied Regions of Apoptosis-Associated Genes

**Figure 3:**
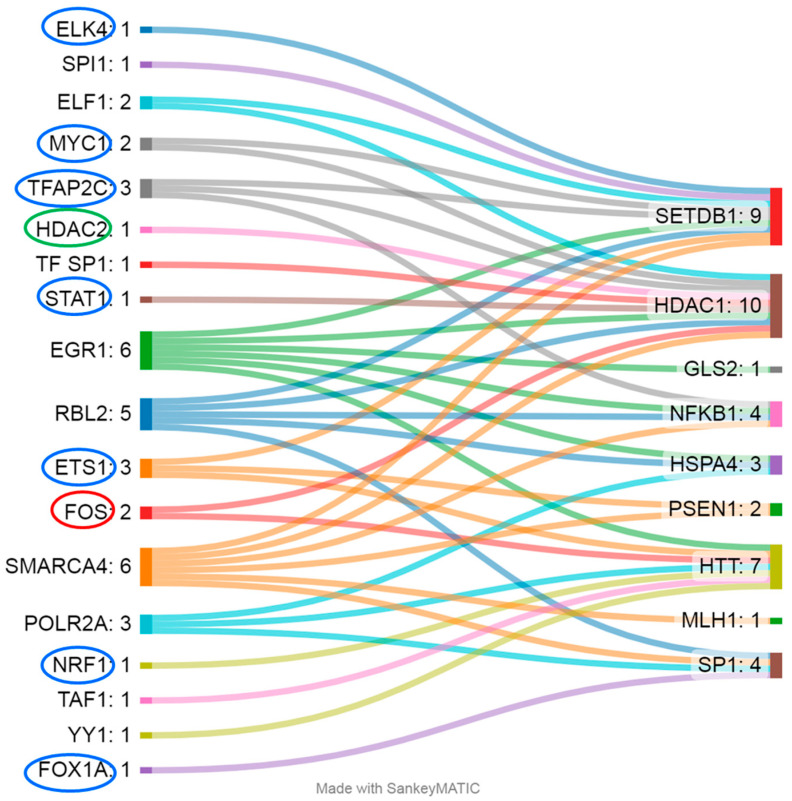
The gene regions (right) in question are regulated by a wide range of TFs (left). Blue ovals indicate TFs whose affinity weakens upon DNA methylation, red ovals denote TFs whose affinity strengthens with the upregulation of genes, and green ovals indicate that the affinity is enhanced but leads to the downregulation of the respective gene.

Most of the differentially methylated CpG sites identified were located within regulatory sequences, potentially indicating their influence on expression. For example, CGIs in *SETDB1*, *TWIST1*, *HSPA4*, *HDAC1*, *NFKB*, *SP1*, *BCL11B*, *MLH1*, and *BDNF* are located in GeneHancer elements [22]; CpG sites in *PSEN1*, *NFKB1*, *HTT*, and *BDNF* are within regions hypersensitive to DNase I [22]; and CpG sites in *PSEN1*, *SETDB1*, *HSPA4*, *HDAC1*, *NFKB1*, *GLS2*, *SP1*, *HTT*, and *MLH1* are localized to TF-binding sites (according to OREGANO [23] and ENCODE3 [24,25]) (Figure 3). The CpG sites in genes *MIR10B*, *GRIA2*, *LEP*, and *APOE* lie outside the regulatory regions; among these genes, only the analyzed region of the *GRIA2* gene is located outside a CGI [22].

Not all TFs that bind to DNA in the analyzed regions are sensitive to CpG methylation (Figure 3); accordingly, their regulatory activity does not depend on changes in the methylation level of the examined CpG sites. For example, for the studied regions of genes *HSPA4* (TFs: RBL2, EGR1, and POLR2A), *GLS2* (TF: EGR1), and *MLH1* (TF: SMARCA4), evidence indicates binding sites only for TFs that are not sensitive to DNA methylation: either methylation does not affect their binding to DNA, or the binding is mediated by other proteins, such as histones [26,27,28]. Methylation of CpG sites weakens the affinity of TFs such as ETS1 (binds to the analyzed regions of *PSEN1*, *SETDB1*, and *HTT*), TFAP2C (*SETDB1*, *HDAC1*, and *NFKB1*), MYC1 (*SETDB1* and *HDAC1*), ELK4 (*SETDB1*), STAT1 (*HDAC1*), FOX1A (*SP1*), and NRF1 (*HTT*); therefore, we can expect a decrease in the expression of these genes [29,30,31,32,33,34,35,36]. Conversely, DNA methylation enhances the binding affinity of the TF FOS, leading to consequent transcription activation [37] of the genes regulated by it (in this case, these are *HDAC1* and *HTT*), and the same is true for TF HDAC2 (*HDAC1*), but in this case, it results in repression of transcription [38]. Most of the above-mentioned DNA methylation–sensitive TFs are expressed at sufficient levels in a wide range of tissues, including whole-blood cells (2.18 to 187.5 transcripts per million (TPM)) [39]. Exceptions are FOXA1 and TFAP2C, which exhibit tissue specificity and are not normally expressed in leukocytes [39].

#### 2.3.1. Changes in the Expression of Apoptosis-Associated Genes in HD

Analysis of available GEO data revealed that in experiments on the Affymetrix platform, a slight change in expression levels in blood cells from HD patients has been documented for 15 of the genes under study (no data were available for *MIR10B*) [datasets GSE24250, GSE8762, and GSE1751] (Table S6). Decreased expression of *HDAC1*, *NFKB1*, *GLS2*, *SP1*, *GRIA2*, *BCL11B*, and *HTT* was recorded in various datasets. Nonetheless, these differences were not statistically significant when compared with the controls. A statistically significant decrease in expression was recorded only for the *LEP* gene (*p*_adj_ = 0.049) (in the dataset GSE1751), but the other two datasets for the same probe (207092_at) showed overexpression of this gene (Table S8). Before correction for multiple comparisons, there was also a significant decrease in the expression of *MLH1* (in dataset GSE24250, *p* = 0.035) and *HTT* and *APOE* (dataset GSE1751, *p* = 0.042 and 0.010, respectively). However, for *HTT* and *APOE*, there were oppositely directed changes in expression in identical probes when different datasets were compared (Table S8).

From an experiment on the Amersham Biosciences platform, data on 12 of our genes of interest were available. HD patients showed statistically significant—with correction for multiple comparisons—reduced expression of *SETDB1* (*p*_adj_ = 0.019), *SP1* (*p*_adj_ = 0.003), *BCL11B* (*p_adj_* = 0.0000007), *HTT* (*p*_adj_ = 0.033), and *APOE* (*p*_adj_ = 0.030) and upregulation of *PSEN1* (*p*_adj_ = 0.003), *APOE* (*p*_adj_ = 0.001), and *MLH1* (*p*_adj_ = 0.001) (Table S8). Notably, the DNA samples were identical between the GSE1751 and GSE1767 datasets. At the same time, unidirectional expression changes (decrease) have been documented on different platforms for genes *SETDB1*, *TWIST1*, *SP1*, *HTT*, *BDNF*, and *APOE* (Table S8).

#### 2.3.2. Changes in Expression of Apoptosis-Associated Genes in LC

Our reanalysis of four datasets (GSE20189, GSE42834, GSE12771/GPL60102, and GSE12771/GPL6097) on the transcriptome of blood cells in LC (Table S6) indicated minor changes in expression compared with controls. Information was obtained using various microarray platforms. According to the Affymetrix platform data, there were expression changes for 12 genes of interest, and the difference was found for five genes (*HSPA4*, *SP1*, *GRIA2*, *DCL11B*, and *MLH1*-p2) (Table S8); however, after correction for multiple comparisons, the differences were not significant.

Data on expression changes in 13 genes of interest are available for the Illumina platform (three versions: Human HT-12 V4.0, Human-6 v1.0, and Human-6 v2.0 Expression BeadChip). In patients with LC, there was a statistically significant—with correction for multiple comparisons—upregulation of the genes *PSEN1* (*p*_adj_ = 0.002), *NFKB1* (*p*_adj_ = 0.023), *SP1* (*p*_adj_ = 0.0004), and *BDNF* (*p*_adj_ = 0.014) and downregulation of genes *BCL11B* (*p*_adj_ = 2.27 × 10^−16^), *MLH1* (*p*_adj_ = 0.006) (GSE42834), and *GLS2* (*p* = 0.045) (GSE12771/GPL60102). Unidirectional changes in expression in different datasets have been documented for only five genes: *GLS2* and *MLH1* expression levels were diminished, and *LEP*, *SETDB1*, and *SP1* expression levels were elevated in LC (Table S8).

#### 2.3.3. A Comparison of Expression Changes of Apoptosis-Associated Genes Between HD and LC

Summarizing the expression data for the two diseases, it should be noted that the most significant changes in expression were recorded for the *BCL11B* gene (Table S8). In both diseases, however, this is downregulation; hence, these changes are of little relevance to the molecular pathogenesis of dystropy. In addition, a unidirectional increase in the expression in HD and LC has been documented for the *PSEN1* gene (Table S8).

**Figure 4:**
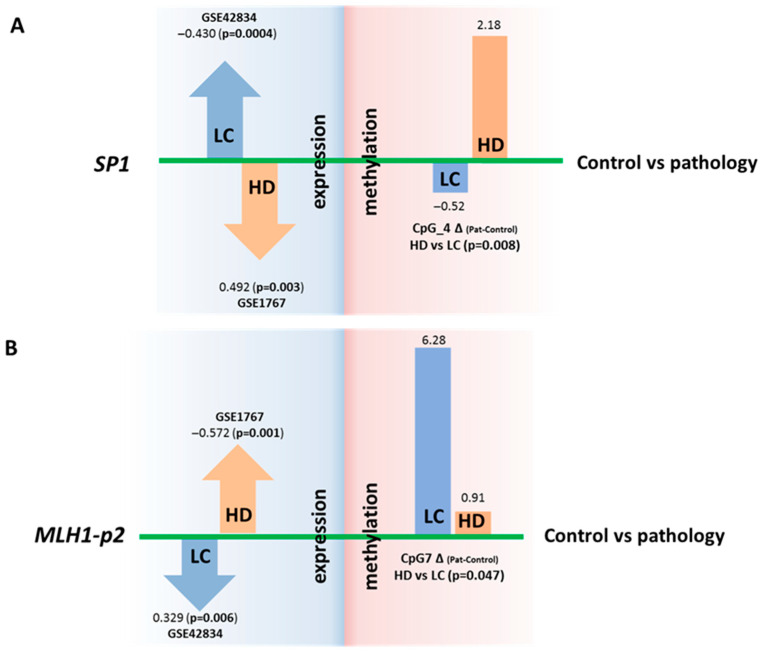
Comparison of DNA methylation levels (in %) of regulatory regions and expression (in logFC) of genes *SP1* and *MLH1*. Changes in *SP1* (**A**) and *MLH1* (**B**) gene expression that are opposite between the two diseases (in relation to controls) and are opposite to changes in methylation of the regulatory regions. The DNA methylation data are from the present study, and the expression data are from the GEO repository.

For two genes (*SP1* and *MLH1*), we noted statistically significant changes in expression that were opposite between the two inversely comorbid diseases when adjusted for multiple comparisons. For instance, *SP1* expression in blood cells is increased in LC and decreased in HD patients. In contrast, the expression of *MLH1* was diminished in LC and elevated in HD (Figure 4, Table S8).

For the *SP1* gene in LC, overexpression (according to our reanalysis of the GEO datasets) corresponds to a decrease (relative to control) in the DNA methylation level of the regulatory region of the gene (our new experimental data), and conversely, in HD, underexpression (data from our reanalysis of the GEO datasets) corresponds to an increase in the DNA methylation level (our new experimental data). For the CpG_4 site, the differences in DNA methylation between HD and LC are statistically significant (*p* = 0.008) (Figure 4 and Figure S1). It has been demonstrated that the whole region, from CpG_3 to CpG_10, binds to RNA polymerase II subunit A, chromatin remodeling factor SMARCA4, and chromatin regulator RBL2, and this event causes, among other things, both promoter-specific chromatin activity and its transcriptional repression [22]. A nucleosomal signal has also been recorded in the CpG_3–CpG_4 region [22]. Our findings suggest that DNA methylation of the studied *SP1* gene region directly affects gene expression; however, the mechanism of this regulation is unclear and requires further research.

In the case of *MLH1*, no oppositely directed changes in DNA methylation levels were noted between the HD and LC groups. A comparison of the data might suggest that the analyzed region of the *MLH1* gene can participate in the regulation of this gene’s expression in cancer but not in neurodegenerative diseases. DNA methylation of the studied region (*MLH1*-p2) may indirectly affect MLH1 expression (Figure 4). The effect on the expression of the second analyzed region (*MLH1*-p1) has been proven and discussed in several studies. Normally, it is demethylated, whereas its methylation at 94–100% completely turns off *MLH1* expression, thereby leading to microsatellite instability, Lynch syndrome, and hereditary predisposition to colorectal, uterine, and genitourinary cancers (CpG_11–CpG_18 are the most crucial) [40,41]. Our work did not reveal any significant increase in DNA methylation in this region in patients with either disease relative to the controls (Table S4).

### 2.4. Interindividual Variation of DNA Methylation Levels

DNA methylation can depend on a large number of factors, one of which is the presence of nucleotide substitutions either in close proximity to or at a considerable distance from the methylated region [42,43,44]. Because BS-NGS makes it possible to detect single-nucleotide polymorphisms (SNPs) (except for C/T substitutions) in the sequenced region, we analyzed the frequency of SNPs and examined their effect on the variation in DNA methylation levels. In total, we identified 17 substitutions with rs# s. Of these, eight (one each in *CYCS*, *EP300*, *MIR2223*, and *NTRK2* and two each in *GRIA2* and *MLH1*-p1) were found in singletons. Therefore, we cannot rule out the possibility that these are random events caused by polymerase errors rather than actual substitutions. In other cases, we performed a selective verification of the identified substitutions.

**Figure 5:**
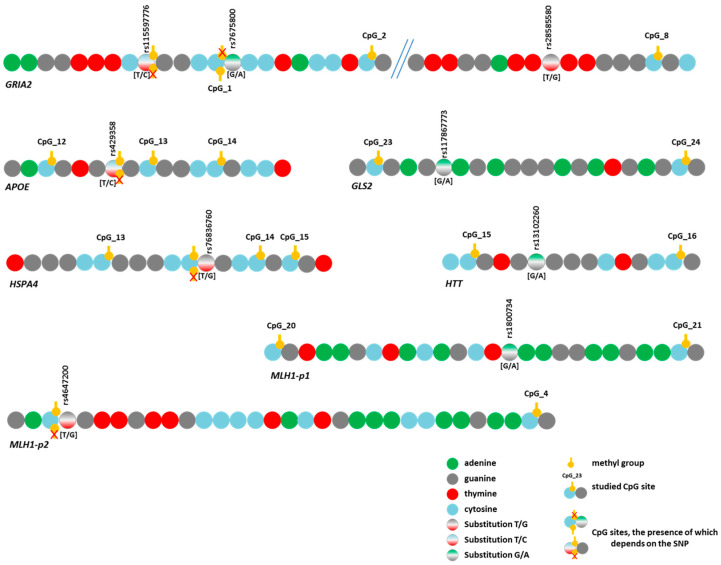
Schematic representation of the location of identified substitutions (SNPs) in the studied gene regions and CpG site locations.

**Figure 6:**
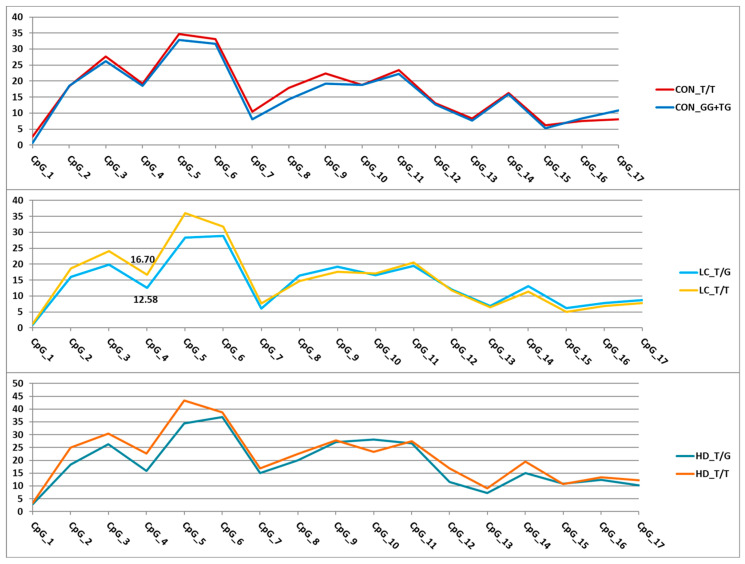
Dependence of methylation levels (in %, *Y*-axis) of the analyzed CpG sites (*X*-axis) in the *GRIA2* gene region on rs28585580 genotypes. For better visualization, the points representing methylation levels in the relevant CpGs are connected by straight lines. Statistically significant differences were found only in the LC group (CpG_4).

For the nine remaining variants (Figure 5), we assessed their effects on DNA methylation levels in the analyzed regions. Associations with the methylation level of CpG sites were detected for eight SNPs (Tables S9 and S10). The trends of variation in DNA methylation levels depending on genotype were similar in different groups. Similar trends were observed for the genes *APOE* and *MLH1*-p2 and the three SNPs in *GRIA2*. As an example, Figure 6 presents graphs of the dependence of methylation levels of the examined CpG sites in the *GRIA2* gene depending on rs28585580 genotypes in the three groups of participants.

The two identified variants represented T/C substitutions (rs429358 in the *APOE* gene and rs115597776 in the *GRIA2* gene). During bisulfite sequencing data analysis, it is impossible to determine with complete certainty whether there was a thymine or an unmethylated cytosine in the original sequence because both bases are read as T. Thus, the detected associations of DNA methylation levels with these SNPs (Tables S9 and S10) may involve not substitutions per se but only methylated cytosines at these positions. However, these SNPs directly affect the possibility of methylation itself because the C allele leads to the emergence of a new CpG site (Figure 5). Regarding rs429358 (in the *APOE* gene), this phenomenon has been emphasized in the literature [45,46], and it has been reported that this SNP affects the level of *APOE* expression through a change in the methylation level of its promoter [47].

**Figure 7:**
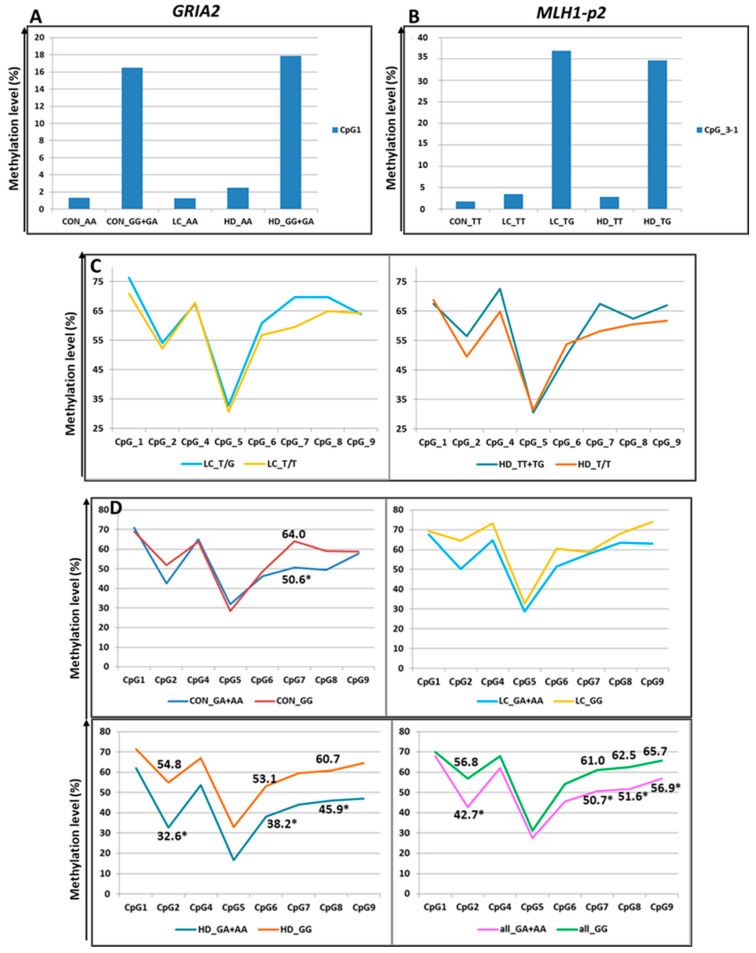
Methylation levels of CpG sites (in %) depending on SNPs genotypes. (**A**) Methylation of CpG_1 in the *GRIA2* gene depending on rs7675800 genotype. (**B**) Methylation of CpG_3-1 (new site) in the *MLH1*-p2 gene depending on rs4647200 genotype (CpG_3 was excluded from association analysis because of low read depth). (**C**) Methylation of the *MLH1*-p2 region depending on rs4647200 genotypes. (**D**): Influence of rs1800734 genotype within the CGI in the *MLH1* promoter on methylation levels of CpG sites (in %) within the CpG shore. For better visualization, the points representing the methylation levels of the relevant CpGs are connected by straight lines. *-the differences are statistically significant.

Three additional SNPs under study also altered the CpG sites. First, rs7675800 in the *GRIA2* gene represents a G/A substitution immediately after cytosine in CpG_1. Accordingly, this CpG site only exists with the G allele of rs7675800. Carriers of the A allele had an extremely low level of CpG_1 methylation, whereas in carriers of the G allele, this level was much higher (the differences were statistically significant in the CON group and in the pooled three groups) (Figure 5 and Figure 7, Table S10).

The SNP rs4647200 in the promoter region of the *MLH1* gene creates a new CpG site (CpG_3-1). In the present study, the CpG site at rs4647200 was found only in the LC and HD groups. The DNA methylation level of the whole region was slightly higher in carriers of the rare allele than in homozygotes for the ancestral allele, but the differences were significant only for CpG_2 (in all groups combined) and CpG_7 (Figure 5 and Figure 7, Table S10). Carriers of the ancestral allele had an extremely low methylation level at the CpG site; in heterozygotes, it was significantly higher; homozygotes for the rare allele were not found in our groups (Figure 7). Previously, we found a similar pattern of methylation of this *MLH1* gene region depending on rs4647200 genotype in patients with atherosclerosis, both in peripheral blood leukocytes and in the carotid arteries (both intact and affected by atherosclerosis) and the great saphenous vein [48].

A new CpG site arose in the analyzed region of the *HSPA4* gene due to a T/G substitution (rs76836760) situated immediately after cytosine (Figure 5). We did not observe methylation at the newly formed sites in our samples. Nevertheless, the methylation level of other CpG sites in the studied region depended on this SNP genotype (Table S10).

The four identified SNPs did not change the structure of CpG sites: rs117867773 in *GLS2*, rs28585580 in *GRIA2*, rs13102260 in *HTT*, and rs1800734 in the first fragment of *MLH1*. Two of them (rs117867773 and rs28585580) have been poorly studied, but variants in *HTT* and *MLH1* have been shown to have pathogenetic significance in the development of HD and cancer, respectively. For example, rs13102260 in the *HTT* gene disrupts the binding of the NF-kB TF to the promoter, thereby reducing gene transcription. In addition, this substitution modifies the pathological effect of the expanded alleles in the *HTT* gene: being at the *cis* position toward the mutation, the A allele is associated with a later age of disease onset, but in the *trans* position, it predisposes to an earlier onset of the disease [49]. However, the effect of this substitution on DNA methylation has not been discussed in the literature.

rs1800734 is frequently investigated due to its impact on methylation levels in the regulatory region of the *MLH1* gene and gene expression. There is evidence of an association between CpG shore methylation levels in the promoter region of *MLH1* with the rs1800734 genotype and an effect of this polymorphism on the microsatellite instability phenomenon in colorectal cancer. This polymorphism is also associated with hepatocellular carcinoma, gastric cancer, lung cancer, and its prognosis [50,51,52,53,54]. We performed an additional search and examined the possible association of rs1800734 (as part of the CGI-containing *MLH1*-p1 region) with the methylation level of CpG sites in the second studied region of the *MLH1* gene (the shore of the same CGI), approximately 1.5 Kb away from rs1800734. We noted lower methylation levels of CpG sites in heterozygotes than in carriers of the ancestral allele (Figure 7), consistent with the data of other researchers [55]. The differences were not statistically significant among all examined sites. Nevertheless, in the CON group, the differences were significant for CpG_7 (*p* = 0.04), and in the HD group, for sites CpG_2 (*p* = 0.03), CpG_6 (*p* = 0.02), and CpG_8 (*p* = 0.03). When the groups were combined, the differences were more distinct for sites CpG_2 (*p* = 0.004), CpG_7 (*p* = 0.01), CpG_8 (*p* = 0.05), and CpG_9 (*p* = 0.02).

## 3. Discussion

In the search for dystro**p**ic genes associated with neurodegenerative diseases and cancer, we investigated the regulatory regions of 23 apoptosis-associated genes. The majority (67%) of the studied regions had extremely low levels of DNA methylation, with the mean level for these sites across all groups not exceeding 3%. All hypomethylated regions are colocalized with CGIs or CGI shores (regions not farther than 2000 bp from a CGI). Accordingly, we noted marked hypomethylation in most of the studied CGIs (15 of 18). Interestingly, CpG sites in the genes *MIR10B*, *LEP*, and *APOE* were located completely within CGIs but had a higher level of methylation. The mean DNA methylation level of the analyzed *MIR10B* region in all groups was 26.8%, for the *LEP* gene, it was 39.8%, and for the *APOE* gene, 95.8%.

To compare DNA methylation levels between groups, we set a 1.5% threshold for changes in methylation levels as a criterion for differential methylation. This choice was due to the extremely low methylation levels at most of the tested CpG sites, located predominantly in a CGI. Accordingly, the range of variation in these regions was extremely small. The differences between the three groups (CON, HD, and LC) more effectively differentiated the HD group from the controls (28 CpG sites in genes SETDB1, TWIST1, HDAC1, GLS2, MIR10B, GRIA2, BCL11B, LEP, and APOE), whereas only seven CpG sites (in the genes PSEN1, GRIA2, LEP, MLH1-p2, and APOE) distinguished the LC group. It is possible that the entire set of analyzed genes contributes to the pathogenesis of HD to a greater extent than to the pathogenesis of LC.

Statistically significant differences in DNA methylation levels between patients with HD and LC were detected at 41 CpG sites in 16 genes. The large number of differences (26 sites) registered for extremely hypomethylated sites is significant. For five genes at individual sites, we found changes in DNA methylation that were opposite between the two groups of patients with dystropic diseases (in relation to controls); the differences between the patient groups were statistically significant (*SETDB1*, *TWIST1*, *HDAC1*, *SP1*, and *GRIA2*) (Table S7). It should be noted that the studied regions of the genes *SETDB1* and *HDAC1*, as well as the two differentially methylated sites of the *TWIST1* gene, were fully hypomethylated. For the differentially methylated CpG sites in *GRIA2* and *SP1*, the mean methylation level ranged from 8.2% to 33.4% (Table S4). At the same time, the range of variation in the DNA methylation level at any of the sites did not exceed 10% (Table S7). It remains to be determined whether differences of this magnitude can affect changes in the expression of the examined genes and contribute to “neurological disease–cancer” dystropy. These five genes are weakly expressed in diverse tissues, and their expression levels in such tissues do not exceed 100 TPM [39].

Since we did not have the opportunity to perform expression analysis of these genes in the same patients, we analyzed the available expression data from open databases. The analysis of differential expression of these genes in the open-access data yielded inconsistent results for three genes: among different samples, there were opposite changes in expression levels of SETDB1 in HD and in the expression of *TWIST1* and *HDAC1* in LC (Table S7). Two of these genes (*SP1* and *GRIA2*) showed differential expression between the two diseases, but there were significant differences in the HD group (or LC group) from the control only in the expression level of the *SP1* gene (Table S8). At the same time, changes in DNA methylation were opposite to changes in expression of the *SP1* gene (in both diseases). These findings indicate the involvement of the studied region of the *SP1* gene in the regulation of its expression and the possible participation of *SP1* in the dystropy between LC and HD.

One of the partner proteins of SP1 is huntingtin, and it is known that the expansion of (CAG)n repeats in the *HTT* gene leads to HD. On the one hand, the expression of the *HTT* gene is regulated by the SP1 TF [56]; on the other hand, it has been reported that huntingtin can bind SP1 [57], and the strength of this interaction depends on the length of the polyglutamine repeat in huntingtin: the longer the repeat, the more strongly it binds to SP1, thereby reducing its bioavailability [57,58]. As a result, the expression of several genes is selectively decreased, but the exact mechanism of this process is unclear [58,59,60]. It has been suggested that the broad pathological effects of mutant huntingtin are due to the specific alteration of its interactions with SP1 [61]. At the same time, *SP1* plays an important role in neoplastic transformation. The expression levels of this TF differ among different tumors and stages of carcinogenesis. It has been shown that SP1 is essential for tumor growth in lung adenocarcinoma; its expression is significantly increased during the neoplastic transformation stage and maintained during the 2nd and 3rd stages, but dramatically declines at the metastasis stage [62]. The mechanism of “HD–LC” dystropy may be as follows: by SP1 binding, mutant huntingtin prevents the upregulation of biologically available SP1 to the level necessary for initiating neoplastic transformation.

We also noticed a dependence of methylation levels of the analyzed CpG sites on SNPs in the tested regions; in some cases, our data were confirmed by the results of other researchers [45,46,47,48,49,50,51,52,53,54].

Our study has several limitations. First, the sample size was small for all three groups. However, with such a rare disease as HD, it is difficult to collect a large group of patients that is homogeneous in other characteristics. We ensured that the HD group was uniform in terms of age and ethnicity. The sample size for the other two groups was similar to that of the HD group. Another limitation was that the effect of SNPs on gene expression was investigated in even smaller samples; therefore, the obtained association data should be considered only as an interesting observation that requires further exploration. Nevertheless, there is independent published evidence on the SNP influence on gene expression, and in addition, two SNPs (rs7675800 and rs4647200) lead to the gain and methylation of new CpG sites. In addition, it should be noted that we did not have the opportunity to analyze gene expression in our patients directly, but we discussed our results by bringing in the expression data for these genes that are available in open data sources.

Of the 596 CpG sites examined, only 90 were present on the Illumina chips (Table S4, column “Illumina ID”). We compared the methylation levels of genes exhibiting the most interesting results (*SETDB1*, *TWIST1*, *HDAC1*, *SP1*, *GRIA2*, and *MLH1*) with publicly available data (Table S11). We performed Pearson’s correlation analysis on a dataset of 37 CpG sites, which were analyzed separately for patients with LC, patients with HD, and healthy controls. This analysis led to several observations:

(1) Even within a single tissue type, the methylation levels of individual CpG sites varied substantially across different studies (Table S11).
(2) This variability generally occurs in the same direction within the same tissue type. The correlation coefficient ranged from 0.47 to 0.95 in normal lung tissue and 0.50 to 0.99 in tumor lung tissue (Figure S2A,B), from 0.63 to 0.99 in PBMCs from HD patients, and was approximately 0.98 in healthy individuals (Figure S2C,D). The methylation levels in cell cultures (GSE147153: “Parental”, “Leaders”, and “Followers”) differed significantly from those in patient tissues.
(3) While average methylation levels differ significantly between tissue types, the pattern of variability remains correlated in most cases. When comparing methylation in whole blood (our study) and lung tissue, the correlation coefficient ranged from 0.2 to 0.7 in cancer tissue and 0.1 to 0.9 in normal tissue (Figure S2A,B). Comparing methylation in the cortex and blood cells yielded correlation coefficients of 0.63–0.96 in HD patients and 0.59–0.97 in healthy individuals (Figure S2C,D).
(4) The degree of correlation varied between genes, with the most stable values observed for the MLH1 gene, for which the largest number of CpG sites were compared.

Therefore, although blood cells do not perfectly reproduce the methylation profile of other tissues, they exhibit a similar pattern of methylation variability and can potentially serve as surrogate markers for methylation studies.

Therefore, despite the limitations, we assume that the results of our study on DNA methylation of apoptosis-associated genes suggest the possible involvement of five genes (*SETDB1*, *TWIST1*, *HDAC1*, *SP1*, and *GRIA2*) in the phenomenon of inverse comorbidity of neurodegenerative diseases and cancers. For these genes, we found that changes in CpG methylation levels were oppositely directed in the two groups of patients ( compared to the control group). For the *SP1* gene, this finding is supported by our analysis of open-access expression data and fits a probable mechanism of “HD–LC” dystropy. Notably, all data (ours and previous) were obtained from peripheral blood leukocytes, thereby opening widely accessible prospects for the development of a biomarker system based on readily available biological samples from patients.

## 4. Materials and Methods

### 4.1. The Studied Groups

Three groups were compiled for the study: (1) patients with a confirmed diagnosis of HD (*n* = 31; male/female ratio (M:F) = 1.1; age 60.5 ± 6.0 years (mean ± SD)), without cancer of any localization, and (2) patients with LC (*n* = 31; M:F = 2.4; age 60.3 ± 6.6 years), whose blood samples were taken before adjuvant chemotherapy, non-smokers, and without neurological diseases (the groups of patients were selected at specialized healthcare institutions; Tables S2 and S3); (3) a control group (CON) consisting of residents of Tomsk city (*n* = 42; M:F = 1.3; age 64.6 ± 6.9 years), who had no obvious symptoms of cardiovascular, oncological, or neurological diseases according to questionnaire data. For all LC patients and controls, the normal length of CAG repeats in the *HTT* gene was confirmed by fragment analysis on an ABI 3730 Genetic Analyzer (Thermo Fisher Scientific, Waltham, MA, USA). All those examined were European.

### 4.2. The Genes Examined

In the first stage of studying the dystropy phenomenon, we reconstructed the associative gene networks of HD involved in apoptosis as a key biological process in cancer pathogenesis. We then selected the highest-priority genes using the ANDSystem tool [10]. We generated a list of genes for analysis by taking into account the functional characteristics of the products of the highest-priority genes and their regulatory relationships (Table S1).

### 4.3. The Analyzed Genomic Regions

We chose target regions for DNA methylation analysis according to the following criteria: (1) the region is located near a gene of interest; (2) the region plays a regulatory role in the gene; (3) the region harbors an enhancer or an active promoter; and (4) the region is characterized by DNA methylation variability according to the ENCODE consortium data (https://www.encodeproject.org (accessed on 2 March 2024)).

### 4.4. The Experiments

Genomic DNA was isolated from peripheral blood leukocytes by phenol–chloroform extraction [63] and treated with sodium bisulfite using the EZ DNA Methylation Kit (Zymo Research, Orange, CA, USA).

Target DNA fragments were amplified using the BioMaster HS-Taq PCR-Color (2×) kit (Biolabmix, Novosibirsk, Russia) with specific PCR primers (Table S4) for each primer pair. The concentration of the amplicons was measured with the Qubit 3.0 fluorimeter (Thermo Fisher, Waltham, MA, USA)

Subsequent analysis was performed using bisulfite amplicon sequencing (BSAS) via massive parallel sequencing, as described by Masser et al. [64]. Amplicons produced by different PCRs from a DNA sample were pooled together in an equimolar ratio. To prepare the libraries, pooled amplicons were purified using the GeneJET NGS Cleanup Kit (Thermo Fisher, Waltham, MA, USA). The concentration of the purified amplicon mix was measured using a Qubit 3.0 fluorimeter (Thermo Fisher, Waltham, MA, USA). DNA libraries were prepared and indexed using either the Nextera XT DNA Library Prep Kit or Nextera DNA Flex Library Prep Kit (Illumina, San Diego, CA, USA). The resulting DNA libraries were sequenced on a MiSeq System (Illumina, San Diego, CA, USA) using the MiSeq Reagent Kit v2 (300 cycles) in the 2 × 150 bp paired-end sequencing mode.

### 4.5. Data Analysis

Initial data analysis was performed using generally accepted methods and pipelines (Table S5). CpG site methylation was quantified as the percentage of reads corresponding to methylated cytosine among all cytosine reads at a specific genomic position.

The distribution of CpG site methylation levels in the groups under study was described as the mean value and standard deviation. Differentially methylated CpG sites were identified via linear regression, where the methylation level was the dependent variable and the independent variables included the ordinal ID number of an experiment, DNA sample, and disease. This approach allowed us to separate interindividual variation (within each group) from systematic errors. We evaluated associations with identified nucleotide substitutions using the Kruskal–Wallis test and Mann–Whitney test (in STATISTICA v.6.0). Differences were considered significant at *p* <0.05.

To analyze differential expression of target genes in peripheral blood leukocytes in HD patients (or LC patients) compared to controls, we used open-access data from the GEO repository (Table S6) and performed the statistical analysis using the built-in GEO2R tool [https://www.ncbi.nlm.nih.gov/geo/ (accessed on 5 June 2024)].

## 5. Conclusions

In this study, we investigated DNA methylation levels in the regulatory regions of 23 apoptosis-associated genes as candidate loci potentially linked to ‘cancer–neurodegeneration’ dystropy. Our findings suggest that five genes (*SETDB1*, *TWIST1*, *HDAC1*, *SP1*, and *GRIA2*) are likely involved in the dystro**p**ic relationship observed between Huntington’s disease and non–small cell lung cancer. We validated this hypothesis for the *SP1* gene by analyzing publicly available data. We discuss the potential mechanisms underlying the ‘cancer–neurodegeneration’ dystropy. Additionally, we analyzed the impact of SNPs on inter-individual variability in DNA methylation levels. Based on our data analysis, we conclude that blood cells exhibit a methylation variability pattern similar to that of other tissues and could therefore serve as surrogate markers in methylation studies.

## Data Availability

Dataset is available upon request from the authors.

## Supplementary Materials

The following supporting information can be downloaded at: https://www.mdpi.com/article/10.3390/epigenomes9030028/s1, Table S1: The studied apoptosis-associated genes; Table S2: Characteristics of patients with Huntington’s disease (HD); Table S3: Characteristics of patients with non-small cell lung cancer (LC); Table S4: Feature of the studied regulatory regions of apoptosis-associated genes; Table S5: Algorithms/Tools/Approaches Used for Bioinformatics Processing of BSAS Results; Table S6: Expression analysis datasets from GEO repository [1]; Table S7: CpG sites significantly different between the studied groups; Table S8: Differential expression of target genes; Table S9: Location of the identified substitutions; Table S10: Associations of methylation level with nucleotide substitutions in the studied regions; Table S11: Methylation microchips data for genes with differently directed methylation relative to control in NSCLC and HD in various tissues (according to open sources); Figure S1. Profiles and patterns of methylation of the studied region of the SP1 gene in the studied groups; Figure S2. Heat map of Pearson’s r-correlation of methylation levels from open datasets and our data.

## Abbreviations

The following abbreviations are used in this manuscript:

HD	Huntington’s disease
LC	non–small cell lung cancer
CON	Control
CGIs	CpG islands
TSS	Transcription start site
TF	transcription factor
*p*_adj_	*p*-value adjusted
SNP	single-nucleotide polymorphism
TPM	transcripts per million
